# The association between music therapy and mental health in Chinese university students: a moderated mediation model of self-evaluation and gender

**DOI:** 10.3389/fpsyg.2025.1746614

**Published:** 2026-02-04

**Authors:** Wei Wang

**Affiliations:** School of Music, Hanshan Normal University, Chaozhou, China

**Keywords:** gender, mental health, music therapy, self-evaluation, university students

## Abstract

This research examines the effects of music therapy participation on mental health among Chinese university students, focusing on the mediating role of self-evaluation and the moderating role of gender. A moderated mediation model was proposed to explain how and for whom music therapy exerts its benefits. A cross-sectional survey was conducted with 1,000 students recruited through multi-stage random sampling from four universities across China. Participants completed the Music Therapy Participation Scale (MTPS), the Self-Evaluation Scale (SES), and the Mental Health Status Questionnaire (MHS-Q). Data were analyzed using structural equation modeling (SEM). Results indicated that higher music therapy participation was significantly associated with better mental health (β = −0.30, *p* < 0.001). Self-evaluation partially mediated this relationship (indirect effect β = −0.11, 95% CI [−0.14, −0.07]). Gender moderated the direct path, with a stronger effect observed for female students (interaction β = 0.09, *p* = 0.002). The findings suggest that music therapy improves mental health both directly and through enhanced self-evaluation, with gender shaping the strength of the direct benefit. These insights support the integration of tailored, gender-sensitive music therapy programs into university mental health services to promote student well-being.

## 1 Introduction

Mental health disorders are an essential issue in the world as the prevalence has been on the increase among the youths and young adult population (World Health Organization [WHO], 2022). A university student population is one of the most vulnerable groups that must manage academic stress, shifts in social spheres, and identity formation (Auerbach et al., 2018). Epidemiological surveys indicate that the problem is widespread among students, ranging from 14% to 22% in the West, and therefore the issue should be approached in its context (Stallman, 2010; Cuijpers et al., 2019).

The problem of mental health among university students is a burning issue in China. As stated, the issue rate was rather average a few years ago (Chen et al., 2013). However, the final surveys show an upward movement with a significant change. According to some of the studies, even in China, as many as 38% of the population of university students have mental health problems, and the percentage of the female population is even higher (Li et al., 2021). Such inconsistencies between studies can be explained by differences in measurement instruments and sampling, as well as by changes in the socio-academic environment over time (Liu et al., 2022). This rising trend demands viable, available, and effective intervention measures.

Music therapy, which is considered the use of musical interventions to achieve therapeutic objectives through clinical and evidence-based practices, has been deemed a safe and effective non-pharmacological methodology (Shultis and Schreibman, 2020). It can help reduce symptoms of anxiety and depression and enhance the overall quality of life across diverse populations, and meta-analytic reviews support these assertions (Aalbers et al., 2017; Leubner and Hinterberger, 2017; de Witte et al., 2020). Nevertheless, the process by which music therapy operates, primarily in the Chinese cultural and educational context, remains unexplored. In addition, as in the case of sustainable and inclusive interventions presented in other sources devoted to the topic, individualization and situational appropriateness of the specified programs, whether in the context of the educational environment (Roldan-Canovas et al., 2024) or the city’s well-being (Ortiz Diaz and Abbas, 2024), are the determinants of the success of any interventions.

The research presents a theoretical framework and applies it to hypothesis testing to understand how and to whom music therapy enhances mental health. Based on existing theories, we assume that self-evaluation, an essential part of self-concept, is the primary psychological process (mediator) that transforms musical engagement into improved mental health (Fancourt and Finn, 2019). At the same time, we test the question of whether gender should be influential (moderate) on the effectiveness of the direct action of music therapy, taking into consideration possible biological, psychosocial, and cultural conditions that can determine the difference in responses (Chen and Zhou, 2022; Tran et al., 2024). Elucidating these pathways, the research is expected to inform the design of more specific and compelling mental health promotion methods for Chinese university students.

## 2 Theoretical framework and hypotheses

### 2.1 Music therapy and mental health: a multi-theoretical perspective

#### 2.1.1 Neurobiological theory

Music engages a broad bilateral network of brain areas associated with emotion, memory, and rewards, and can regulate autonomic functions (Koelsch, 2014; Zatorre, 2018). Such neural activity can promote neural plasticity, balance stress hormone levels (e.g., cortisol), and improve heart rate variability, thereby decreasing physiological arousal and increasing emotional well-being (Bernardi et al., 2006; Chanda and Levitin, 2013; Trost and Frühholz, 2021).

#### 2.1.2 Psychological theory

Music can be considered one of the best means of emotional manipulation and expression (Juslin and Västfjäll, 2008; Carlson et al., 2021). The negative affective states, anxiety and sadness, are reduced because of listening to music and the music involvement. It is possible to enhance mental health through mechanisms such as mood congruence, cognitive distraction, and the evocation of positive affect.

#### 2.1.3 Sociological theory

Music is commonly communal. Music-making in the group may result in social bonding, loneliness, social anxiety, and perceived social support, all of which are vital toward the preservation of mental health (Pearce et al., 2015; Williams et al., 2021). It aligns with studies highlighting the importance of social connectedness and supportive conditions in reducing stress and burnout (Tran et al., 2024).

### 2.2 The mediating role of self-evaluation

Self-evaluation, which includes constructs such as self-esteem and self-efficacy, is the subjective assessment of personal value and potential (Rosenberg, 1965). It is a strong predictor of mental health, in which low self-evaluation is a predictive risk factor of depression and anxiety (Sowislo and Orth, 2013; Orth and Robins, 2022). We suggest that music therapy improves self-assessment in a variety of ways: (a) learning musical skills will help in developing a sense of competence and self-efficacy (Geeves et al., 2020); (b) aesthetic engagement and expressing artistic ideas can increase self-worth; and (c) positive feedback in a therapeutic or group context will validate the individual (MacDonald et al., 2021). Such enhanced self-assessment then leads to improved mental health. Therefore, self-evaluation is theorized to serve as an intermediary.

### 2.3 The moderating role of gender

The prevalence and response of mental health to interventions vary between genders. Psychological distress is frequently reported among female university students in China, which may be explained by the combination of biological factors, gender-specific social norms, and peculiarities of stressors (Li et al., 2021; Chen and Zhou, 2022). Also, studies indicate that women might also be more emotionally responsive and receptive to aesthetic and interpersonal stimuli, such as those found in music (Linnemann et al., 2015; Park et al., 2021). Thus, we also hypothesize that the effect of gender would mediate the association between music therapy and mental health, with a stronger association among female students.

### 2.4 Research hypotheses

H1: Mental health is positively linked to music therapy participation.

H2: Self-assessment has a positive relationship with mental health.

H3: Self-assessment mediates the connection between engagement in music therapy and mental well-being.

H4: There is a moderator effect by gender on the direct association between music therapy attendance and mental health, whereby the correlation is higher amongst female students.

## 3 Research methods

### 3.1 Participants and procedure

A cross-sectional study was done in September 2025. Participants were recruited using the multi-stage random sampling method (Kish, 1965). First, four universities in the various geographical regions of China (East, South, West, North) were randomly selected from a national list. Second, across each university, academic departments were randomly selected. Finally, based on the student rosters of assigned departments, individual students were randomly invited to participate. This strategy aimed to improve representativeness while accounting for the practical limitations of a nationwide random sample (Babbie, 2020).

The first web survey produced 1020 responses. Twenty responses were excluded: 12 for completing the study for less than 1/3 of the median time (indicating inattentive responding), and 8 for identical responses to all of the items. The final sample was 1,000 valid responses (98.04% retention rate). *A priori* power analysis in G*Power 3.1 with a linear multiple regression (fixed model, R^2^ increase), effect size, f^2^ = 0.05, α = 0.05, and the power of 1 −β = 0.95 showed a total sample size of 758 (Faul et al., 2009). Our final sample was above this requirement. All participants gave electronic informed consent. This study was approved by the Ethics Committee of Hanshan Normal University (Approval No. 2025111901).

### 3.2 Measures

All the instruments were Chinese versions that had been validated and were reliable among the required populations.

#### 3.2.1 There will be participation in music therapy (independent variable)

Participation in music therapy will be assessed using a 12-item Music Therapy Participation Scale (MTPS), which was developed specifically for the present study in accordance with AMTA guidelines (2020). Items assessed frequency and depth of engagement in structured music-based activities with therapeutic intent over the past 6 months (e.g., “I have participated in group music therapy sessions,” “I use prescribed music listening for relaxation”). Responses were on a 5-point Likert scale (1 = Never, 5 = Very Often). Higher scores indicated greater participation. The scale demonstrated excellent internal consistency in this sample (Cronbach’s α = 0.92).

#### 3.2.2 Self-evaluation (mediator)

Measured using the 10-item Self-Evaluation Scale (SES), a widely used Chinese adaptation of the Rosenberg Self-Esteem Scale (Wang and Zhang, 2011). Items (e.g., “On the whole, I am satisfied with myself”) are rated on a 4-point Likert scale (1 = Strongly Disagree, 4 = Strongly Agree). Higher scores indicate more positive self-evaluation. Cronbach’s α was 0.86.

#### 3.2.3 Mental health (dependent variable)

Evaluated based on 12 questions on the Mental Health Status Questionnaire (MHS-Q), a screening instrument that is validated to be used on Chinese young adults (Chen and Zheng, 2021). They will include the past 2 weeks with the symptoms of depression, anxiety, and stress. Responses will be on a 4-point Likert scale (1 = Never, 4 = Almost Always). Primary analysis was performed using a continuous total score, with higher scores indicating poorer mental health (i.e., more symptoms). A cut-off score of ≥24 (including the study in the validation sample) and above (which falls in the top quartile of the score range) was adopted for descriptive purposes to determine the “probable cases” (Chanda and Levitin, 2013; Chen and Zheng, 2021). This yielded a case prevalence of 25.5% in our sample, which is similar to recent epidemiological estimates. Cronbach’s α was 0.88.

#### 3.2.4 Demographic variables

Information regarding gender (male/female), age, annual family income (categorized as Low: <¥50,000; Medium: ¥50,000–150,000; High: >¥150,000), and parents’ marital status (married/not married) was collected.

### 3.3 Data analysis

Data were analyzed using the software SPSS 27.0 and AMOS 27.0. First, descriptive statistics and bivariate Pearson correlations were calculated. To test H1 and H2, an initial linear regression analysis was performed. To examine the fitted integrated moderated mediation model (H3 and H4), we used SEMs with the maximum likelihood estimation (Kline, 2023). The model incorporated music therapy as a predictor variable, self-evaluation as a mediator variable, mental health (continuous score) as an outcome variable, and gender as a moderator of the direct relationship of music therapy to mental health. Model fit was assessed using the χ^2^/df ratio (<3), CFI (>0.95), TLI (>0.95), and RMSEA (<0.06) (Hu and Bentler, 1999). The significance of the indirect effect (mediation) was tested using bias-corrected bootstrap confidence intervals (5,000 samples) (Hayes, 2022). A significant interaction term (Music Therapy × Gender) would indicate moderation, followed by a simple slope analysis to probe the effect at each gender level.

## 4 Results

### 4.1 Descriptive statistics and sample characteristics

A cross-sectional survey was administered to a final sample of 1,000 Chinese university students, recruited via a multi-stage random sampling procedure. First, four universities were randomly selected from different geographical regions of China (East, South, West, North). At each university, several academic departments were chosen at random, and then individual students were randomly selected from the lists of those departments. There were 1,020 respondents in the first pool. Ten responses were dropped: 12 because their time spent in completing those responses was less than a third of the median time (indicating that they were not paying attention), and 8 because they gave the same response to all items (suggesting that they were patterned responding). This left a final valid sample of 1,000 participants (retention rate: 98.04%).

As seen in Table 1, the mean age of the sample was 20.02 years (SD = 1.05), and the gender distribution was nearly equal (51.4% female). The probable mental health problems were determined based on the MHS-Q cutoff score (≥24) and affected 25.5% of cases (*n* = 255). The chi-square tests demonstrated significant correlations of the status of the mental health problems with several key variables including gender (χ^2^ = 11.89, *p* < 0.001, with more women having the issues), level of music therapy participation (χ^2^ = 55.35, *p* < 0.001), level of self-evaluation (χ^2^ = 19.58, *p* = 0.036) and the category of family income (χ^2^ = 6.64, *p* = 0.036). Parental marital status and age had no significant correlation with the status of mental health problems in this analysis.

**TABLE 1:** Sample demographic characteristics (*N* = 1000).

Item	Category	All participants (*n* = 1000) *n* (%)	No probable MH problems (*n* = 745) *n* (%)	Probable MH problems (*n* = 255) *n* (%)	χ^2^	*p*
Gender	Male	486 (48.6)	385 (51.7)	101 (39.6)	11.892	**0.001**
	Female	514 (51.4)	360 (48.3)	154 (60.4)		
Age group	18–20 years	721 (72.1)	531 (71.3)	190 (74.5)	10.832	0.093
	21–26 years	279 (27.9)	214 (28.7)	65 (25.5)		
Mean age (SD)		20.02 (1.05)	20.06 (1.04)	19.89 (1.07)	–	–
Family income	Low (<¥50k/year)	442 (44.2)	321 (43.1)	121 (47.5)	6.642	0.036*
	Medium (¥50–150k/year)	460 (46.0)	357 (47.9)	103 (40.4)		
	High (>¥150k/year)	98 (9.8)	67 (9.0)	31 (12.2)		
Parents’ marital status	Married	935 (93.5)	700 (94.0)	235 (92.2)	1.223	0.269
	Not married	65 (6.5)	45 (6.0)	20 (7.8)		
Music therapy participation	Low (Quartile 1)	250 (25.0)	220 (29.5)	30 (11.8)	55.350	**<0.001**
	Moderate (Q2 & Q3)	500 (50.0)	367 (49.3)	133 (52.2)		
	High (Quartile 4)	250 (25.0)	158 (21.2)	92 (36.1)		
Self-evaluation level	Low (Quartile 1)	250 (25.0)	202 (27.1)	48 (18.8)	19.584	**<0.001**
	Moderate (Q2 & Q3)	500 (50.0)	377 (50.6)	123 (48.2)		
	High (Quartile 4)	250 (25.0)	166 (22.3)	84 (32.9)		

*p < 0.05. Bold values indicate statistically significant differences or coefficients (*p* < 0.05).

### 4.2 Reliability and validity

All measurement instruments demonstrated good to excellent reliability and structural validity in the current sample (Table 2). Factor loadings for all items exceeded 0.60, Cronbach’s α values were above 0.70, and KMO values surpassed 0.75, indicating the suitability of the data for factor analysis and subsequent modeling.

**TABLE 2:** Reliability and validity of measurement scales (*N* = 1000).

Variable	Item	Factor loading	Cronbach’s α	KMO
Music therapy (MTPS)	MT1	0.72	0.92	0.91
	MT2	0.78		
	MT3	0.81		
	MT4	0.84		
	MT5	0.86		
	MT6	0.85		
	MT7	0.82		
	MT8	0.79		
Self-evaluation (SES)	SE1	0.69	0.86	0.80
	SE2	0.75		
	SE3	0.73		
	SE4	0.80		
	SE5	0.82		
	SE6	0.83		
	SE7	0.64		
	SE8	0.69		
	SE9	0.84		
	SE10	0.71		
Mental health (MHS-Q)	MH1	0.63	0.88	0.76
	MH2	0.73		
	MH3	0.91		
	MH4	0.76		
	MH5	0.59		

### 4.3 Correlation analysis

Bivariate Pearson correlations among the key continuous variables are presented in Table 3. As hypothesized, music therapy participation was significantly and negatively correlated with mental health problems (*r* = −0.31, *p* < 0.001), indicating that higher involvement was associated with fewer reported symptoms. Self-evaluation was also significantly and negatively correlated with mental health problems (*r* = −0.37, *p* < 0.001). Furthermore, participation in music therapy was positively correlated with self-evaluation (*r* = 0.42, *p* < 0.001). These preliminary correlations provided support for testing the proposed mediation model.

**TABLE 3:** Pearson correlations among key continuous variables (*N* = 1000).

Variable	M	SD	1	2	3
1. Music therapy	2.98	0.89	–	–	–
2. Self-evaluation	2.85	0.54	0.42*** (*p* < 0.001)	–	–
3. Mental health problems	22.70	6.72	−0.31* (*p* = 0.018)	−0.37* (*p* = 0.008)	–

*p < 0.05, ***p < 0.001.

### 4.4 Testing the moderated mediation model

To test the hypothesized model–where self-evaluation mediates the link between music therapy and mental health, and gender moderates the direct path–we employed structural equation modeling (SEM) using maximum likelihood estimation.

#### 4.4.1 Model fit

The proposed model demonstrated an acceptable to good fit to the data: χ^2^/df = 2.89, Comparative Fit Index (CFI) = 0.96, Tucker-Lewis Index (TLI) = 0.94, Root Mean Square Error of Approximation (RMSEA) = 0.05 (90% CI [0.04, 0.06]), Standardized Root Mean Square Residual (SRMR) = 0.04. These indices suggest that the model adequately represents the observed relationships in the data.

#### 4.4.2 Path coefficients and hypotheses testing

**FIGURE 1:**
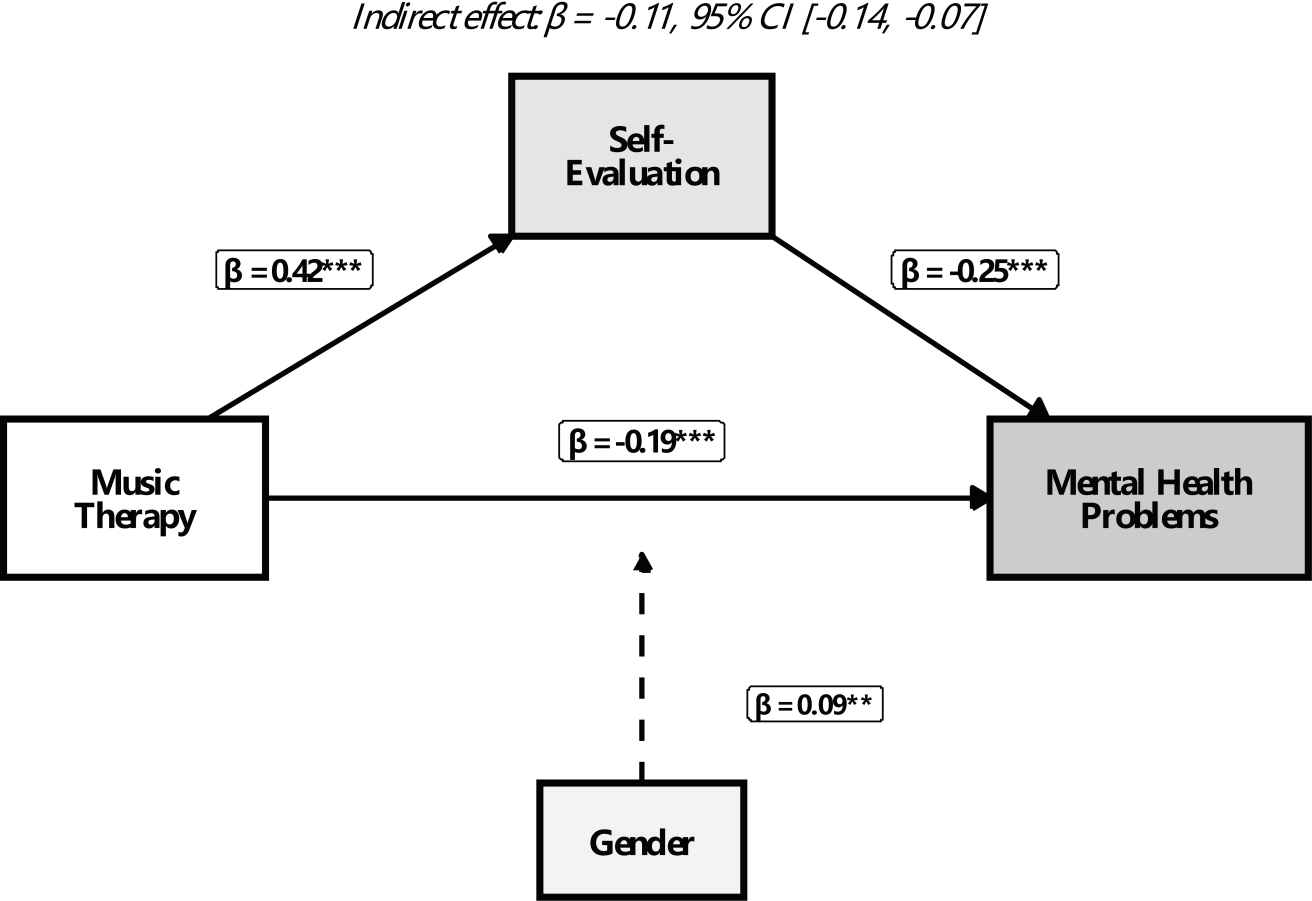
Final moderated mediation model with standardized path coefficients. **p < 0.01, ***p < 0.001. Dashed line indicates moderation path.

The standardized path coefficients are illustrated in Figure 1 and detailed in Table 4.

**TABLE 4:** Structural equation modeling (SEM) results for the moderated mediation model.

Path (relationship)	β (std. coeff.)	S.E.	*Z*-value	*p*	95% BC Boot CI
**Direct effects**					
Music therapy → self-evaluation	0.42	0.03	12.81	**<0.001**	[0.36, 0.48]
Self-evaluation → mental health problems	−0.25	0.04	−6.92	**<0.001**	[−0.32, −0.18]
Music therapy → mental health problems (direct effect)	−0.19	0.04	−5.12	**<0.001**	[−0.26, −0.12]
**Interaction effect**					
Music therapy × gender → mental health problems	0.09	0.03	3.08	**0.002**	[0.03, 0.15]
**Indirect effect**					
Music therapy → self-evaluation → mental health problems	−0.11	0.02	–	**<0.001**	[−0.14, −0.07]

*Bold values indicate statistically significant differences or coefficients (*p* < 0.05).

#### 4.4.3 Direct and mediating effects

Music therapy participation significantly predicted higher self-evaluation (β = 0.42, *p* < 0.001). Self-evaluation, in turn, significantly predicted lower levels of mental health problems (β = −0.25, *p* < 0.001). The direct effect of music therapy on mental health problems, after accounting for the mediator, remained significant and negative (β = −0.19, *p* < 0.001). The indirect effect of music therapy on mental health problems through self-evaluation was statistically significant (β = −0.11, 95% Bias-Corrected Bootstrap CI [−0.14, −0.07]). This confirms that self-evaluation partially mediates the relationship between music therapy and mental health, supporting H3. The total effect of music therapy on mental health problems was β = −0.30 (*p* < 0.001), supporting H1. The positive association between self-evaluation and mental health (fewer problems) supports H2.

#### 4.4.4 Moderating effect of gender

**FIGURE 2:**
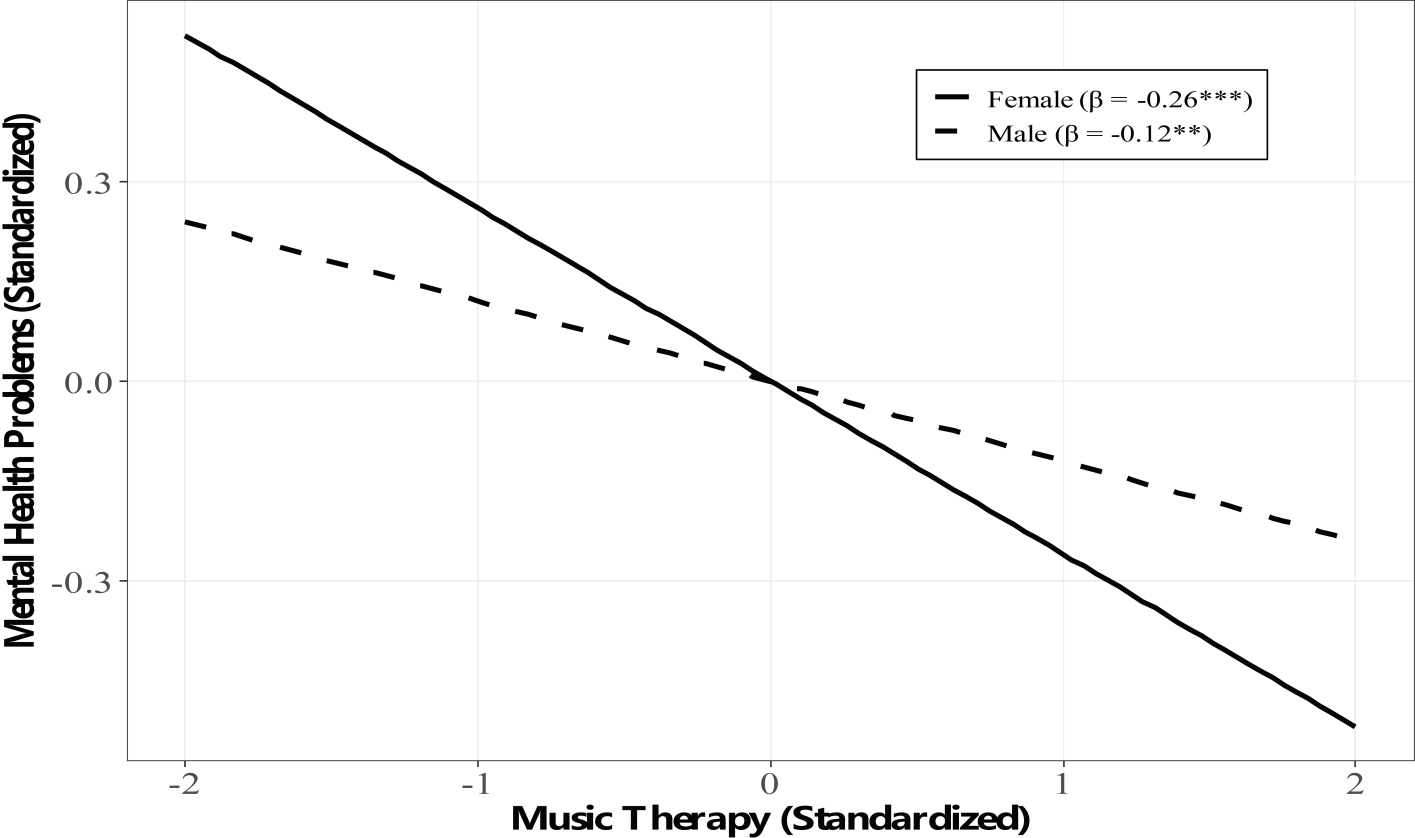
Simple slopes for the moderating effect of gender on the direct path. *p < 0.05, ***p < 0.001.

The interaction term between music therapy and gender on mental health problems was significant (β = 0.09, *p* = 0.002). Simple slope analysis (Figure 2) revealed that the adverse effect of music therapy on mental health problems was significant for both males (β = −0.12, *p* = 0.001) and females (β = −0.26, *p* < 0.001), but the effect was significantly more substantial for female students. This finding supports H4, indicating that gender moderates the direct path.

### 4.5 Ancillary analysis: family income and mental health

An exploratory analysis of variance (ANOVA) was conducted to further examine the unexpected relationship between family income and mental health problem status identified in the chi-square test (see Table 5). Results indicated a significant main effect of income category on mental health problem scores, *F*(2, 997) = 3.58, *p* = 0.028. *Post hoc* comparisons using Tukey’s HSD revealed that the Medium-income group reported significantly fewer mental health problems than the Low-income group (*p* = 0.045). The high-income group was no different from the Low or Medium groups. It is non-linear, indicating that moderate economic resources, in the case of Chinese university students, are associated with minimal mental health risk, which could be explained by the absence of sufficient support and excessive performance-related pressure, as in the high-income group.

**TABLE 5:** One-way ANOVA and *post hoc* comparisons for mental health problems by family income category.

Family income category	*n*	Mental health problem score M (SD)	95% confidence interval	Tukey HSD *post hoc* comparisons
Low	442	23.10 (1.20)	[22.98, 23.22]	vs. Medium: *p* = 0.045
Medium	460	21.45 (1.10)	[21.33, 21.57]	vs. Low: *p* = 0.045
High	98	22.13 (1.30)	[21.87, 22.39]	vs. Low: *p* = 0.471 vs. Medium: *p* = 0.682

*p < 0.05.

## 5 Discussion

This research examined a mediated mediation model linking music therapy participation and mental health among Chinese university students. The results are a solid argument for the proposed framework. First, as hypothesized, greater involvement in music therapy was associated with improved mental health (fewer reported symptoms). Second, self-evaluation also enhanced this relationship and was confirmed as a mediating mechanism. Third, the direct effect of music therapy was moderated by gender, and female students obtained a more direct mental health benefit as a result of participation.

### 5.1 The mediating pathway: enhancing the self

The substantial intermediary effect of self-evaluation aligns with psychological assumptions that therapeutic practices promote mastery and self-esteem (Sowislo and Orth, 2013). With competence, creativity, and positive reinforcement in a therapeutic environment, music therapy is likely to offer powerful experiences of competence and validation (Fancourt and Finn, 2019; Geeves et al., 2020). Such improved self-assessment is then mobilized as an internal strength that shields against stress and negative affect, thereby increasing mental health (Orth and Robins, 2022). This process highlights the importance of the fact that the positive effects of music therapy are not limited to the short-term mood improvement but extend to a more enduring cognitive-affective shift.

### 5.2 Gender as a moderator: cultural and psychological nuances

The biological and psychosocial viewpoints are combined in the observation that the direct mental health benefits of music therapy were more pronounced in women. We found that women reported more mental health issues as baseline, which is also in line with the national trends (Li et al., 2021). It would render them more accepting of the emotional and communal essence of the music therapy due to their vulnerability to emotional and relational stimuli (Linnemann et al., 2015; Park et al., 2021). Moreover, within the Chinese set-up where women can feel the stress of society, music therapy can serve as a safe and viable means of emotional and stress discharge, which women can readily sympathize with, rather than with their male counterparts (Chen and Zhou, 2022).

### 5.3 Unexpected pattern of family income

The negative conclusion about family income is worth viewing with caution. Although riches tend to generate protective factors, in high-performing settings, wealth may also be associated with enormous pressures to perform, which may offset its advantages (Luthar et al., 2020). It may be that the “moderate” income group can be financially safe enough without the high-stress, status-seeking demand that leads to anxiety. Here, the link between socioeconomic elements and mental well-being in competitive societies is multi-dimensional as well, and reflects contemporary literature on sustainability by vindicating the need to address well-being holistically and take into account contextual stressors (Ortiz Diaz and Abbas, 2024).

### 5.4 Implications for practice

#### 5.4.1 University mental health programming

Music therapy-related interventions (e.g., group drumming, therapeutic choirs, guided music listening) should be considered among the options in university student wellness programs because they are non-stigmatizing and engaging (de Witte et al., 2020).

#### 5.4.2 Gender-sensitive intervention

Practitioners are advised to be mindful of the potentially greater direct efficacy of the music therapy process for female students and to accommodate their needs by designing or promoting groups, and by evaluating how to engage male students (MacDonald et al., 2021).

#### 5.4.3 Focus on self-development

Interventions aimed at developing self-assessment should explicitly enhance it through musical exercises and structured positive feedback based on success, and capitalize on this central process to bring about permanent change (Geeves et al., 2020).

### 5.5 Limitations and future directions

This research is limited in several ways. First, its cross-sectional nature does not allow for making definitive causal conclusions. Although our model is conceptually motivated, longitudinal or experimental research is required to establish the direction of effects and to rule out reverse causality (e.g., better mental health leading to increased therapy participation). Second, the self-report scale is prone to biases. Future study may include behavioral observations, physiological indicators, or clinician ratings. Third, the sampling, however, was not entirely nationally representative. More comprehensive random sampling frames should be used in future research. Fourth, we have not quantified the particular forms or “doses” of music therapy; the effectiveness of the various modalities remains a significant future question (de Witte et al., 2022). Lastly, music preference was initially intended to be measured, but was then dropped due to measurement limitations. The development and inclusion of this variable in future work should also be rigorously tested to assess whether it moderates the efficacy of the intervention (Carlson et al., 2021).

## 6 Conclusion

This research contributes to expanding knowledge about the mechanisms of music therapy and the people it directly affects in the Chinese university environment. The findings go beyond merely establishing a relationship between a variable (self-evaluation) and another (gender) by determining that self-evaluation is a fundamental psychological process and that gender is the primary moderating variable. Although cross-sectional data have limitations, the findings strongly indicate that music therapy is a potential field for positively influencing mental health among students (Aalbers et al., 2017; de Witte et al., 2020). The fact that it has two practical purposes, a direct emotional rewards and a means of enhancing the self, makes it a valuable tool. It is advised that universities and mental health professionals create and apply music-based interventions, considering gender differences and students’ emphasis on self-evaluation as a long-term tool for well-being.

## Data Availability

The datasets presented in this study can be found in online repositories. The names of the repository/repositories and accession number(s) can be found in the article/supplementary material.
